# Mn(iii), Fe(iii) and Zn(ii)-serum albumin as innovative multicolour contrast agents for photoacoustic imaging†

**DOI:** 10.1039/d3na00843f

**Published:** 2023-12-24

**Authors:** Enza Di Gregorio, Angelo Scarciglia, Alessandro Amaolo, Giuseppe Ferrauto

**Affiliations:** a Department of Molecular Biotechnology, Molecular Imaging Center, University of Torino Via Nizza 42 10126 Torino Italy enza.digregorio@unito.it +39 0116708459

## Abstract

Here we propose innovative photoacoustic imaging (PAI) contrast agents, based on the loading of Mn(iii)-, Fe(iii)- or Zn(ii)-protoporphyrin IX in serum albumin. These systems show different absorption wavelengths, opening the way to multicolor PA imaging. They were characterized *in vitro* for assessing stability, biocompatibility, and their optical and contrastographic properties. Finally, a proof of concept *in vivo* study was carried out in breast cancer bearing mice, to evaluate its effectiveness for cancer imaging.

## Abstract 2

Here we propose innovative photoacoustic imaging (PAI) contrast agents, based on the loading of Mn(iii)-, Fe(iii)- or Zn(ii)-protoporphyrin IX in serum albumin.

## Graphical abstract

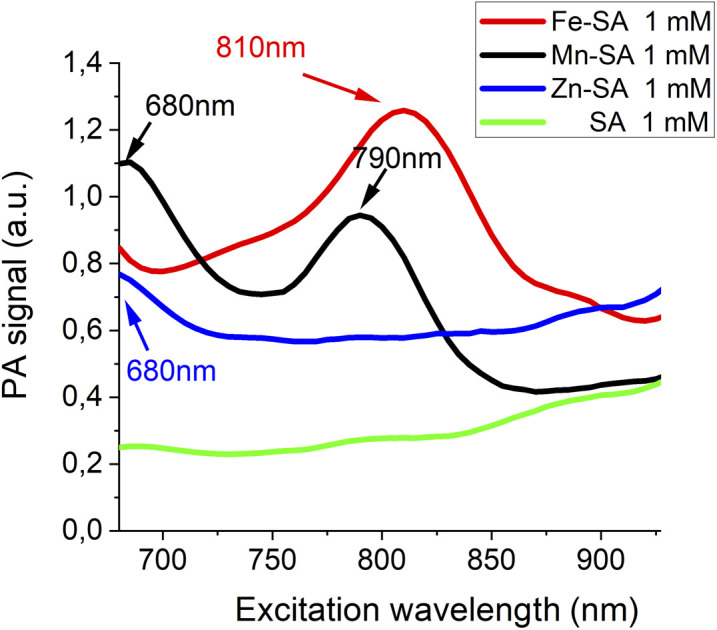

Photoacoustic imaging (PAI) has emerged in the last few years as a useful and innovative technique for biomedical imaging that advantageously combines some properties of light and sound.^1–5^ PAI displays good deep-tissue penetration and spatial resolution (as ultrasound imaging, US) and high sensitivity and possibility of exploiting multiple imaging probes (as optical imaging, OI).

PAI contrast agents (CAs) are excited using pulsed laser light and emit US waves, which can be detected using a typical US transducer to create the image.^6–9^ To be PAI-detectable, a molecule or a supramolecular adduct must fulfil two essential conditions, *i.e.*, (i) rapidly absorb light energy (high molar extinction coefficient, *ε*) and (ii) relaxing mainly through non-radioactive mechanisms instead of radioactive ones (*i.e.*, low quantum yield *Φ* and low emission of fluorescence). Energy will be dissipated by non-radiative mechanisms, such as heating, causing thermoelastic expansion to generate US-waves.^10–12^

In the last two decades, several endogenous and exogenous molecules have been described as suitable PAI CAs.^6–8,13^ For instance, haemoglobin^14–16^ and melanin^16–18^ have been widely exploited as natural PAI CAs to gather physiological information in healthy and pathological tissues. These molecules allow the assessment of vascular volume, oxygen content, perfusion, and other relevant parameters.^19–21^

More recently, the field of PAI research has witnessed the emergence of exogenous contrast agents as a prominent area of investigation. The numerous PAI CAs already proposed fall into four main categories: (i) molecular dyes, classically used in optical imaging (*e.g.* methylene blue, Congo red, near-infrared fluorescent molecules, and indocyanine green),^3,22,23^ (ii) fluorescent proteins (*e.g.* GFP protein^24^), (iii) plasmonic surface resonance noble metal nanoparticles (*e.g.* gold or copper nanoparticles)^24–26^ and (iv) non-plasmonic nano- or microparticles (*e.g.* ICG-loaded mesoporous silica nanoparticles^27^).

PAI CAs should be characterized by biological compatibility, non-toxicity, water solubility, a sufficiently long half-life, and total elimination from the body. Additionally, they should exhibit high light-to-ultrasound energy conversion, be amenable to chemical modifications for targeted applications, be easy to prepare and be stable.

Considering these properties, herein we propose the use of serum albumin (SA)-derived CAs for PAI. These probes are based on the encapsulation of metal porphyrin inside SA.^28,29^

SA-based contrast agents for imaging have garnered significant attention in scientific research due to their potential usefulness, mainly because SA is the most abundant blood protein (concentration of *ca.* 0.65 mM) with a high stability. It exhibits excellent biocompatibility and low toxicity, making it suitable for use in medical applications. Moreover, it has a long circulatory half-life and can be easily modified to load imaging probes. Furthermore, albumin-based contrast agents can specifically accumulate in tumour regions by the enhanced permeability and retention (EPR) effect.^30^

Porphyrins possess unique optical properties that make them ideal candidates for PAI imaging.^31–34^ Their characteristic absorption and fluorescence properties can be finely tuned through structural modifications, enabling efficient light-to-sound energy conversion and enhancing their PA contrast-generating capabilities. Moreover, porphyrins exhibit strong absorption in the near-infrared (NIR) region, where biological tissues have lower absorption, resulting in deeper tissue penetration and improved imaging depth. However, they exhibit a very high cell and tissue toxicity, hampering their application *in vivo* in free form. Therefore, to design a suitable PAI probe, Mn-, Fe-, or Zn-porphyrin were proposed to be loaded inside natural SA.

It has already been demonstrated that SA is able to bind to the heme group constituting the so-called serum heme-albumin, and thanks to its peculiar features, it was exploited as a CA for MRI.^28,29^ The heme group present in haemoglobin is constituted by the Fe(iii)-protoporphyrin IX, Fe-PTP IX^35^ (chemical structure in Scheme 1A).

**Scheme 1:**
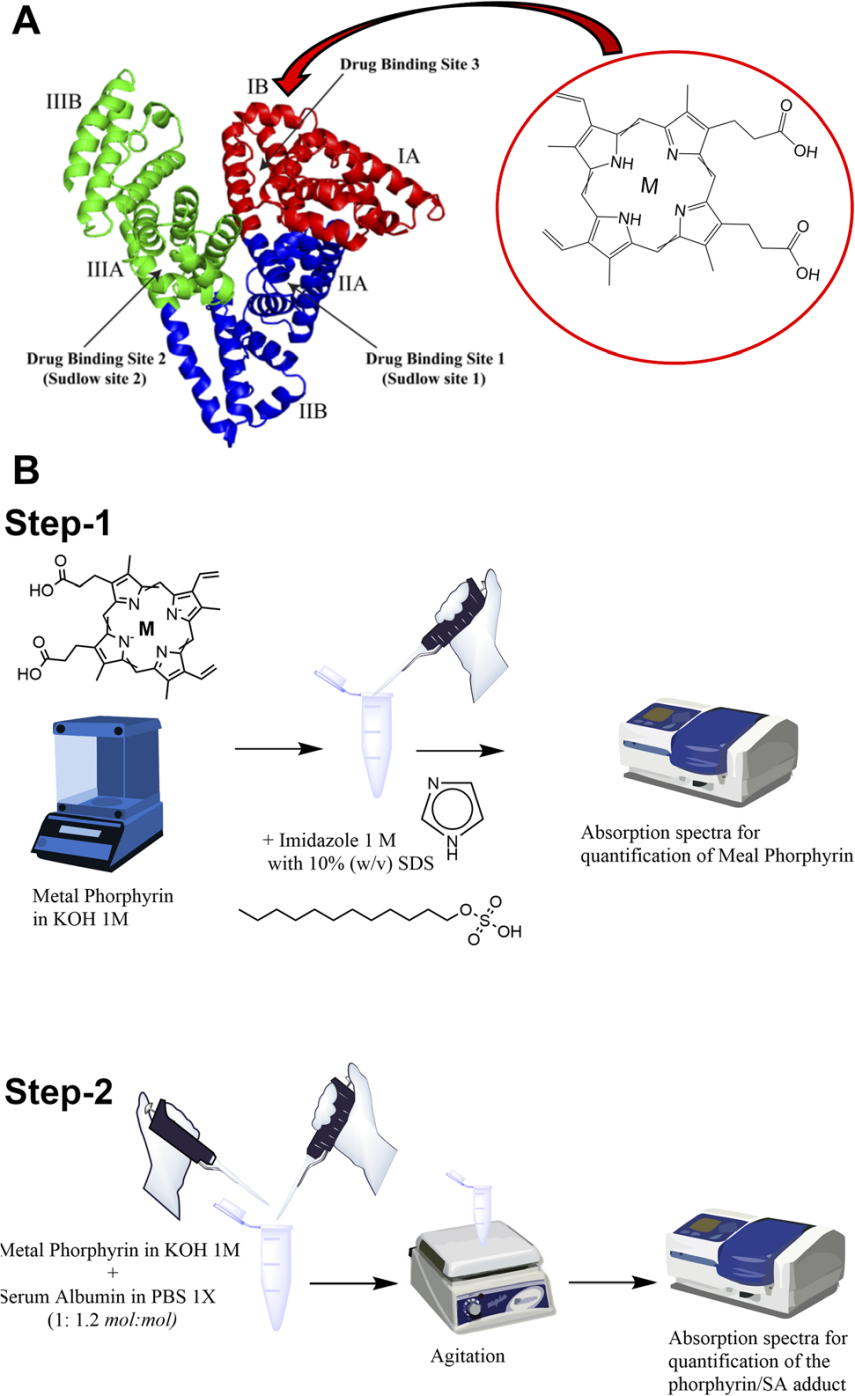
(A) Schematic illustration of the interaction of serum albumin with protoporphyrin IX (M = Mn(iii) or Fe(iii) or Zn(ii)). (B) Scheme of the preparation of metal porphyrin serum albumins (step-1: UV-vis spectrophotometric quantification of metal porphyrin; step-2: preparation and quantification of metal porphyrin serum albumins).

As previously reported, the binding site for the heme group is represented by the subdomain IB corresponding to FA1 (Scheme 1A inset).^36,37^

In this work, three supramolecular adducts were prepared, *i.e.*, (i) Fe(iii)-protoporphyrin IX-loaded SA (hereinafter named Fe-SA), (ii) Mn(iii)-protoporphyrin IX-loaded SA (hereinafter named Mn-SA) and (iii) Zn(ii)-protoporphyrin IX-loaded SA (hereinafter named Zn-SA).

The metal porphyrin hydroxide (0.1 N KOH) was slowly added to SA phosphate-buffered solution (PBS 1X). The concentration of metal porphyrin hydroxide solution was measured by UV-vis spectrophotometry (Scheme 1B step-1; ESI, Fig. S1†). An excess of SA (1 : 1.2 mol : mol) was used to ensure the absence of free toxic porphyrin. The pH of the solution was checked and adjusted to be 7.2 ± 0.1. Solution was gently stirred for 10 min at room temperature. Finally, the preparation was filtered by using a 0.2 μm nitrocellulose filter and stored at 4 °C before further use (Scheme 1B step-2).

The obtained porphyrin-SAs were quantified by spectrophotometric measurements (absorption at 402 nm for Fe-SA, 466 nm for Mn-SA and 416 nm for Zn-SA, Fig. S2†).

The three SA adducts were investigated using photoacoustic imaging technology. PAI and spectra of Mn-SA, Fe-SA and Zn-SA samples were determined on a VisualSonics Vevo 2100 LAZR Imaging Station (VisualSonics, Inc., Toronto, Canada). PA spectra were acquired in the 670–950 nm range. All PA images were co-registered with grey scale B-mode ultrasound images, acquired using a high-frequency ultrasound probe (MS550D, VisualSonics, Canada, broadband frequency: 22 MHz–55 MHz, image axial resolution: 40 μm) at 40 MHz. The specimens' resulting PA spectra are reported in Fig. 1.

**Fig. 1:**
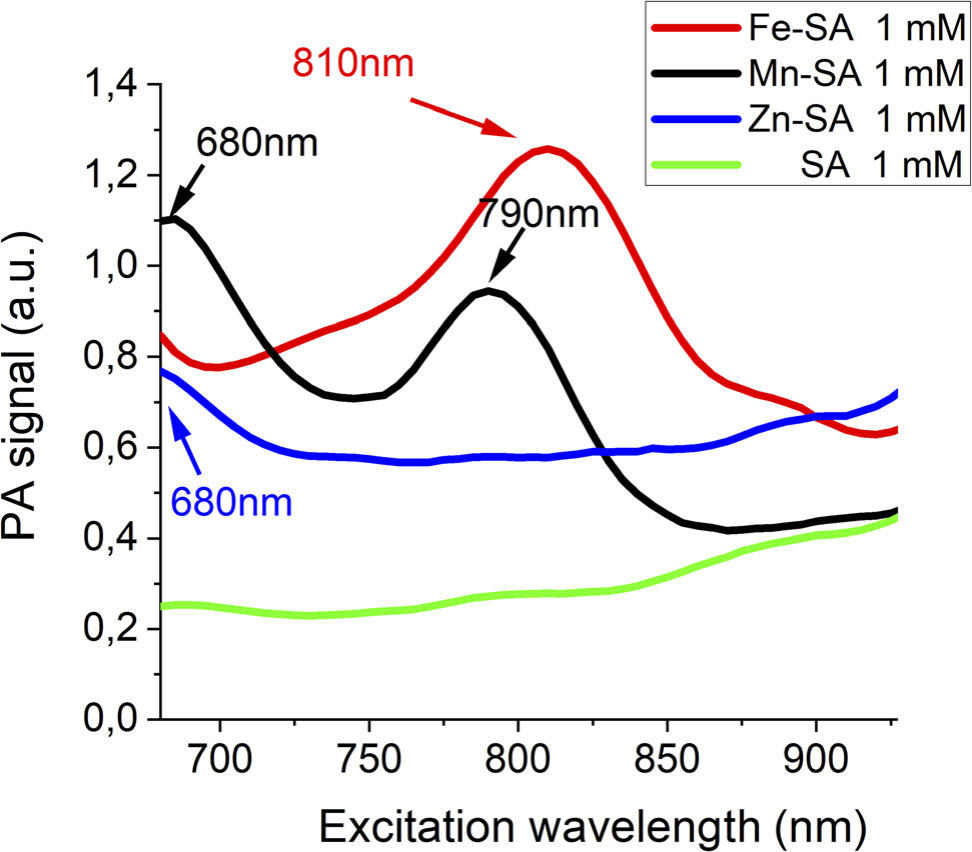
Photoacoustic spectra of Fe-SA, Mn-SA and Zn-SA.

Free serum albumin was used as a control, and it does not display any appreciable PA signal. Only a very broad background signal is present for wavelengths higher than 900 nm (green lines in Fig. 1). PA spectra of Fe-SA (red line Fig. 1) show a peculiar PA peak upon excitation at 810 nm (red line Fig. 1); PA spectra of Mn-SA show two peaks at 680 and 790 nm, respectively (black line Fig. 1); PA spectra of Zn-SA show a PA peak at 680 nm (blue line Fig. 1).

The generation of a PA effect is a consequence of the absorption of light energy in the red/near infrared (NIR) region which is partially re-emitted with non-radiative mechanisms (as thermoelastic expansion). For each supramolecular adduct (i) size, (ii) ζ-potential, and (iii) molar extinction coefficient (*ε*) were measured. Data are reported in Table 1.

**Table 1:** Features of the different supramolecular adducts

	Fe-SA	Mn-SA	Zn-SA	Empty SA
Size	7.6 ± 0.4 nm	7.2 ± 0.3 nm	7.5 ± 0.2 nm	7.0 ± 0.1 nm
ζ-Potential	−8.6 ± 0.4 mV	−8.0 ± 0.2 mV	−7.5 ± 0.2 mV	−7.3 ± 0.3 mV
*ε* (for the quantification of metal-SA adducts)	*λ* = 402 nm, *ε* = 107 mM^−1^ cm^−1^	*λ* = 466 nm, *ε* = 28 mM^−1^ cm^−1^	*λ* = 415 nm, *ε* = 298 mM^−1^ cm^−1^	n.a.
PA peak(s)	810 nm	680 nm, 790 nm	680 nm	n.a.
PA detection threshold	0.08 mM	0.04 mM, 0.04 mM	0.07 mM	n.a.

PA spectra at variable concentrations of Fe-SA in buffer (up to 1 mM) are reported in Fig. 2.

**Fig. 2:**
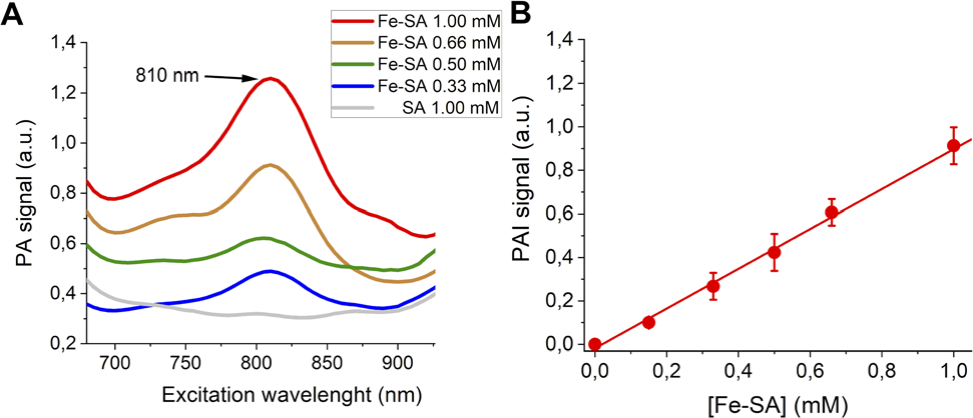
(A) PA spectra of Fe-SA at variable concentrations. (B) Linear correlation of the normalized PAI signal and [Fe-SA].

A PA peak at 810 nm is present. This peak is, as expected, at the same wavelength as the isosbestic point of haemoglobin.^38^Fig. 2B reports the PA effect at 810 nm against the concentration. A linear effect is present, with the detection threshold at *ca.* 0.08 mM.

PA spectra at variable concentrations of Mn-SA in buffer (up to 1 mM) are reported in Fig. 3A. Two PA peaks are present. The first one is at a wavelength lower than 700 nm (centred at *ca.* 680 nm), whereas the second one is at 800 nm.

**Fig. 3:**
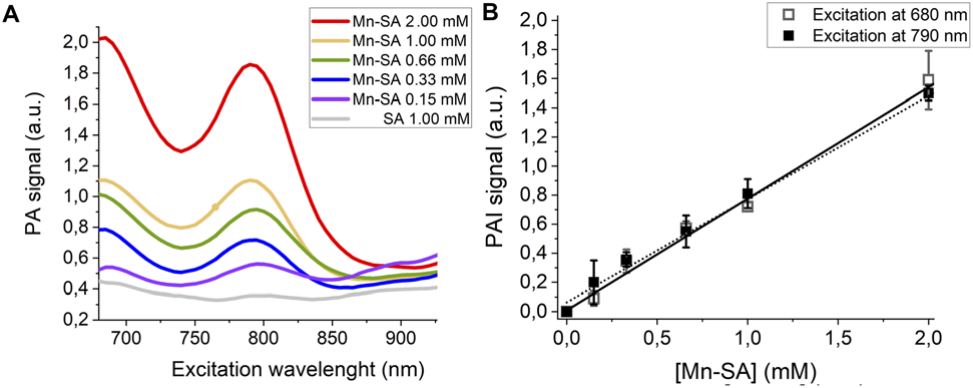
(A) PA spectra of Mn-SA at variable concentrations. (B) Linear correlation of the normalized PAI signal and [Mn-SA].

Fig. 3B reports the PA effect at 680 and 810 nm against the concentration. In both cases a linear effect is present, with a detection threshold at *ca.* 0.04 mM for both the wavelengths.

Finally, PA spectra of variable concentrations of Zn-SA in buffer (up to 1 mM) are reported in Fig. 4A. It has to be noted that for Zn-SA the PA spectra do not display a well-defined but a very broad peak. Despite this, upon excitation, the PA signal at a wavelength of 700 nm linearly correlates with the concentration of Zn-SA.

**Fig. 4:**
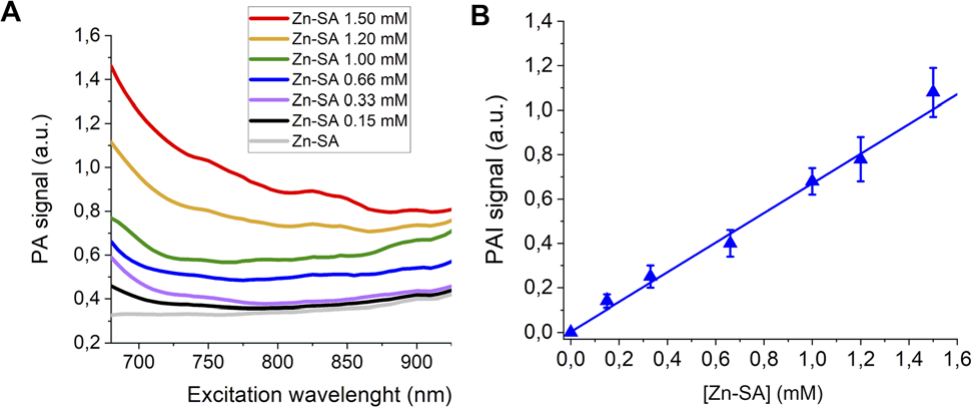
(A) PA spectra of Zn-SA at variable concentrations. (B) Linear correlation of the normalized PAI signal and [Zn-SA].

Fig. 4B reports the PA effect at 700 nm against the concentration. A linear effect is present, with the detection threshold at *ca.* 0.07 mM.

Proof of concept of the feasibility of distinguishing two differently labelled metal-serum albumin was assessed *in vitro* by mixing Zn-SA with Fe-SA.

These two systems have been chosen because of the nonoverlapped PAI signal. In Fig. 5, US and PAI images upon excitation at 700 nm and 800 nm, respectively, are reported.

**Fig. 5:**
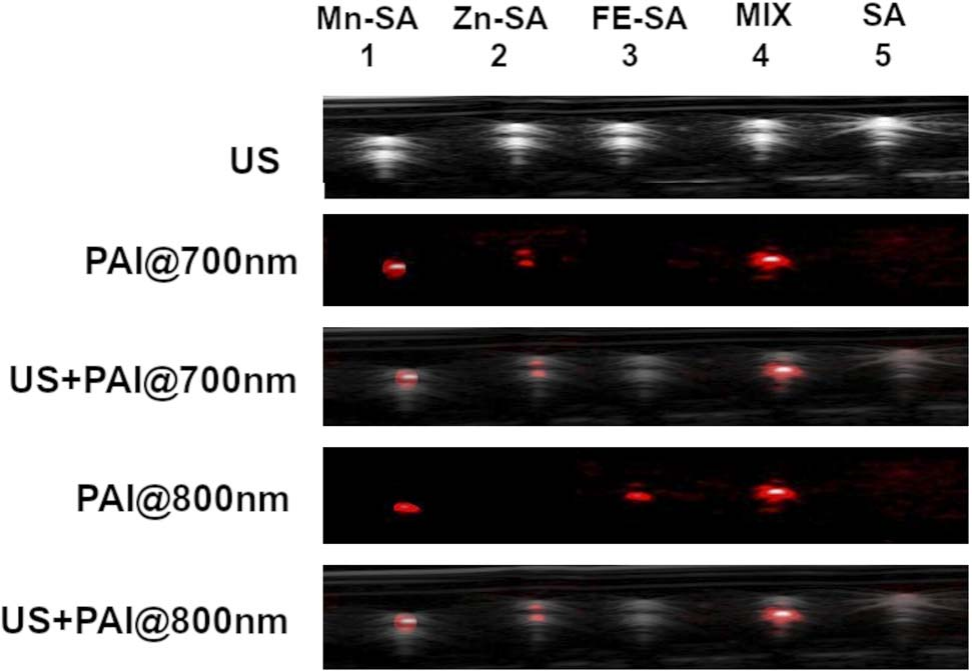
US and PA images (excitation at 700 nm or 800 nm) of plastic tubes filled with Mn-SA, Zn-SA, Fe-SA, a mixture of Zn-SA and Fe-SA or empty SA.

The first tube was filled with Mn-SA, the second one with Zn-SA, the third one with Fe-SA, the fourth tube with a mixture of Zn-SA and Fe-SA and the fifth tube with metal free-SA, as a control.

PA contrast at 800 nm is present in the tubes containing Mn-SA, or Fe-SA, or in the one containing the mixture of Zn-SA and Fe-SA. Conversely, the PA contrast at 700 nm is only present in the tubes containing Mn-SA, Zn-SA, and Zn-SA plus Fe-SA. The tube containing SA-control is not contrasted neither upon excitation at 700 nm nor at 800 nm (considering setting an appropriate threshold for background signals). Hence, Fe-SA and Zn-SA can be distinguished by choosing the appropriate excitation wavelength (800 nm or 700 nm, respectively), and their co-presence in the same tube is highlighted by the double contrast at the two wavelengths.

Stability of the developed metal porphyrin serum albumins was monitored for one week, at 37 °C under agitation. Both the sizes, the ζ-potential and the visible absorbance showed to be constant over time (Fig. S3†).

Furthermore, the supramolecular adducts are not toxic for cells, as assessed by an MTT-assay carried out on murine breast cancer TS/A cells and on murine macrophages J774A.1 cells (Fig. 6). These cell lines were chosen for assessing toxicity since the further *in vivo* study was carried out on a transplantable breast cancer murine model and macrophages are the cells strongly coming into contact with exogenous nanometric adducts or particles.

**Fig. 6:**
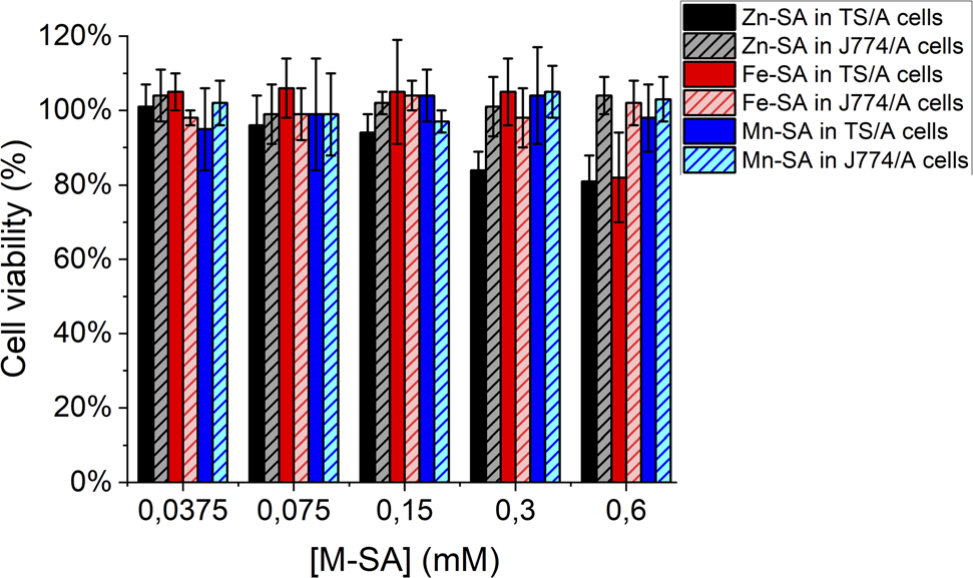
Cell viability assay (MTT test) for murine breast cancer TS/A cells or murine macrophages J774A.1 cells.

As reported, only a little toxicity is present for high concentrations of Zn-SA in TS/A cells.

Zn-porphyrins were reported to be less photostable than their metal-free or other metal-chelated analogues, showing a larger rate of self-bleaching with respect to porphyrins containing other metal centres. This phenomenon is accompanied by a decrease in absorbance and an increase in ROS formation. Tentatively, the decreased cell viability reported for cells upon incubation in the presence of Zn-SA could be accounted for by singlet oxygen generation and Zn-based photoproducts, due to reactions with solvent and oxygen containing species.^39^

Finally, BALB/c mice were inoculated under the skin with 3 × 10^5^ TS/A breast cancer cells. After 10 days, when tumours reached a volume of *ca.* 150 mm^3^, they were used for PA imaging. For this purpose, Fe-SA preparation was i.v. injected in the tail vein (single bolus of 200 μL) at a dose of 0.02 mmol per kg b.w., corresponding to *ca.* 0.2 mM of Fe-SA in the blood.

Representative US (Fig. 7A) and PA images (Fig. 7B–D) of the tumour region upon excitation at 800 nm were reported. In the PA image acquired before the Fe-SA administration (Fig. 7B) only a slight PA signal is present, due to the endogenous presence of blood hemoglobin. Immediately after the administration of Fe-SA (*t* = 5 min), the PA signal at 800 nm was strongly enhanced (Fig. 7C). At *t* = 15 min from the injection the PA signal decreased; however, it was still detectable (Fig. 7D). In the manually drawn ROI in Fig. 7, the contrast enhancement at 5 min was 104 ± 12% and reduced to 48 ± 7% at 15 min.

**Fig. 7:**
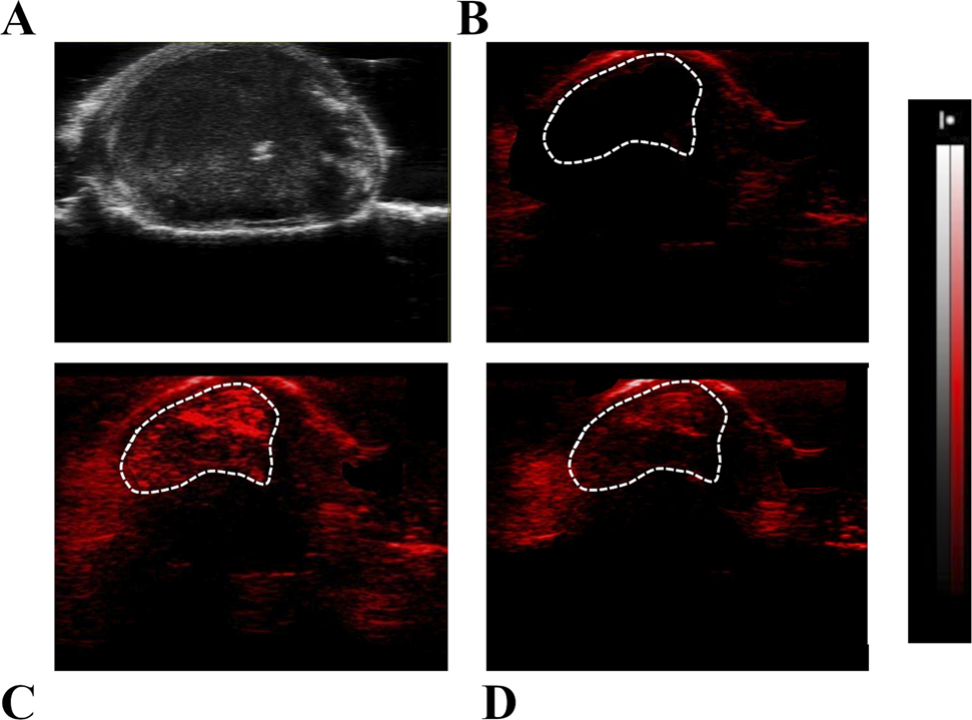
Representative US (A) and PA images with *λ* = 800 nm (B–D) of tumour regions in transplantable murine models; (B) pre-PA image; (C) PA image 5 min and (D) 15 min after i.v. injection of Fe-SA preparation.

The contrast is a consequence of the extravasation of the labelled albumins in the extravascular/extracellular tumour space due to the enhanced permeability and retention (EPR) mechanism, which typically occurs in leaky endothelium and under high oncotic pressure in solid tumours.

## Conclusions

In this work serum albumin was loaded with Mn-, Zn- or Fe-protoporphyrin IX. These systems appear stable and not toxic to cells. They display a peculiar PA effect (at 810 nm for Fe-SA, at *ca.* 700 and at 800 nm for Mn-SA and *ca.* 700 nm for Zn-SA) that makes it possible to distinguish the single contribution when simultaneously present in the region under analysis.

Mn-SA appeared to be more sensitive than Fe-SA and Zn-SA. Finally, we reported as a proof of concept the feasibility of the investigated Fe-SA probe to be visualized *in vivo*. The presence of a significant PA contrast in the tumour region upon i.v. injection of Fe-SA was demonstrated. This suggests the feasibility of developing innovative PAI contrast agents based on porphyrins and their conjugation with serum albumin, merging the contrastographic properties of porphyrins with the biocompatibility of serum albumin.

## Author contributions

Conceptualization, funding acquisition, methodology, resources, supervision and writing-original draft: G. F. and E. D. G.; investigation, data curation and writing – review & editing: E. D. G., A. A., A. S., and G. F.

## Conflicts of interest

There are no conflicts to declare.

## Supplementary Material

NA-006-D3NA00843F-s001

## References

[cit1] Biswas D., Roy S., Vasudevan S. (2022). Micromachines.

[cit2] Jiang H. , Photoacoustic Tomography, CRC Press, 2018

[cit3] Xiao P., Liang M., Yang S., Sun Y., Li J., Gu Z., Zhang L., Fan Q., Jiang X., Wu W. (2023). Biomaterials.

[cit4] Gargiulo S., Albanese S., Mancini M. (2019). Contrast Media Mol. Imaging.

[cit5] Ntziachristos V. (2010). Nat. Methods.

[cit6] Weber J., Beard P. C., Bohndiek S. E. (2016). Nat. Methods.

[cit7] Luke G. P., Yeager D., Emelianov S. Y. (2012). Ann. Biomed. Eng..

[cit8] Pan D., Kim B., Wang L. V., Lanza G. M. (2013). Wiley Interdiscip. Rev.: Nanomed. Nanobiotechnol..

[cit9] Fu Q., Zhu R., Song J., Yang H., Chen X. (2019). Adv. Mater..

[cit10] Liu N., Mishra K., Stiel A. C., Gujrati V., Ntziachristos V. (2022). Adv. Drug Delivery Rev..

[cit11] Razansky D. and Ntziachristos V., in Molecular Imaging in Oncology, ed. O. Schober, F. Kiessling and J. Debus, Springer International Publishing, Cham, 2020, vol. 216, pp. 155–187

[cit12] Attia A. B. E., Balasundaram G., Moothanchery M., Dinish U. S., Bi R., Ntziachristos V., Olivo M. (2019). Photoacoustics.

[cit13] Maturi M., Locatelli E., Monaco I., Comes Franchini M. (2019). Biomater. Sci..

[cit14] Taylor-Williams M., Spicer G., Bale G., Bohndiek S. E. (2022). J. Biomed. Opt..

[cit15] John S., Hester S., Basij M., Paul A., Xavierselvan M., Mehrmohammadi M., Mallidi S. (2023). Photoacoustics.

[cit16] Gao X., Chen X., Hu H., Wang X., Yue W., Mu J., Lou Z., Zhang R., Shi K., Chen X., Lin M., Qi B., Zhou S., Lu C., Gu Y., Yang X., Ding H., Zhu Y., Huang H., Ma Y., Li M., Mishra A., Wang J., Xu S. (2022). Nat. Commun..

[cit17] Li T., Jing W., Fu W., Yan Z., Ma Y., Li X., Ji H., Zhang R. (2023). Biomater. Adv..

[cit18] Liopo A., Su R., Oraevsky A. A. (2015). Photoacoustics.

[cit19] Di Gregorio E., Ferrauto G., Gianolio E., Lanzardo S., Carrera C., Fedeli F., Aime S. (2015). ACS Nano.

[cit20] Ferrauto G., Di Gregorio E., Dastrù W., Lanzardo S., Aime S. (2015). Biomaterials.

[cit21] Quiros-Gonzalez I., Tomaszewski M. R., Golinska M. A., Brown E., Ansel-Bollepalli L., Hacker L., Couturier D.-L., Sainz R. M., Bohndiek S. E. (2022). Cancer Res..

[cit22] Lu C.-H., Hsiao J.-K. (2021). Tzu Chi Med. J..

[cit23] Gonzalez E. A., Lediju Bell M. A. (2022). J. Biomed. Opt..

[cit24] Silva A. D., Serpa C., Arnaut L. G. (2019). Sci. Rep..

[cit25] Pan D., Pramanik M., Senpan A., Allen J. S., Zhang H., Wickline S. A., Wang L. V., Lanza G. M. (2011). FASEB J..

[cit26] Zhang R., Kiessling F., Lammers T., Pallares R. M. (2023). Drug Delivery Transl. Res..

[cit27] Ferrauto G., Carniato F., Di Gregorio E., Botta M., Tei L. (2019). Nanoscale.

[cit28] Fasano M. (2003). J. Inorg. Biochem..

[cit29] Fasano M., Baroni S., Vannini A., Ascenzi P., Aime S. (2001). JBIC, J. Biol. Inorg. Chem..

[cit30] Wu J. (2021). J. Pers. Med..

[cit31] Liu Z., Li H., Tian Z., Liu X., Guo Y., He J., Wang Z., Zhou T., Liu Y. (2022). ChemPlusChem.

[cit32] Fathi P., Pan D. (2020). Nanomedicine.

[cit33] Pu K., Shuhendler A. J., Jokerst J. V., Mei J., Gambhir S. S., Bao Z., Rao J. (2014). Nat. Nanotechnol..

[cit34] Shao S., Rajendiran V., Lovell J. F. (2019). Coord. Chem. Rev..

[cit35] Joniová J., Kažiková V., Gerelli E., Bánó G., Wagnières G. (2018). J. Biomed. Opt..

[cit36] Ascenzi P., Fasano M. (2009). IUBMB Life.

[cit37] Mishra V., Heath R. J. (2021). Int. J. Mater. Sci..

[cit38] Zhou Y., Yao J., Wang L. V. (2016). J. Biomed. Opt..

[cit39] Golec B., Buczyńska J., Nawara K., Gorski A., Waluk J. (2023). Photochem. Photobiol. Sci..

