# Supplementary material for: Mn(iii), Fe(iii) and Zn(ii)-serum albumin as innovative multicolour contrast agents for photoacoustic imaging
Source: Nanoscale Adv. 2023 Dec 24;6(3):777–81. doi: 10.1039/d3na00843f (PMC10825928; doi:10.1039/d3na00843f)
Supplement: NA-006-D3NA00843F-s001 [file NA-006-D3NA00843F-s001.pdf]

Electronic Supplementary Material (ESI) for Nanoscale Advances.
This journal is © The Royal Society of Chemistry 2023

Electronic Supporting Information (ESI)
for

Mn(III), Fe(III) and Zn(II) - Serum Albumin as innovative multicolour contrast agents for Photoacoustic Imaging

Enza Di Gregorio,*[a] Angelo Scarciglia, [a] Alessandro Amaolo [a] , and Giuseppe Ferrauto [a]

[a] Molecular Imaging Center, Department of Molecular Biotechnology, University of Torino, Via Nizza 42, 10126, Torino (It),
Phone: 0039-01167078459
enza.digregorio@unito.it

Content

- *Materials and Methods*
- *Supporting results*
- *Supporting references*

Materials and Methods

<u>*Chemicals*</u>

Mn (III) Protoporphyrin IX (PTP IX) chloride was purchased from SCBT - Santa Cruz Biotechnology, Inc. Fe (III) Protoporphyrin IX chloride, Zn (II) Protoporphyrin IX chloride, Bovine Serum Albumin (BSA) and all other chemicals were purchased from Sigma St. Louis, Mo., USA and used without further purification.
Mn(III), Fe(III) and Zn(II) PTP IX were dissolved in a KOH 1N solution and stored frozen (-20°C). The porphyrin concentration was spectrophotometrically determined by using a 6715 UV-Vis Spectrophotometer Jenway (Bibby Scientific Limited, Beacon Road, Stone, Staffordshire, ST15 OSA, UK). This measurement was carried out by adding the metal porphyrin to a 10% (*w/v*) SDS solution in 1M Imidazole (for Mn (III) PTP IX Abs_{555nm} ε=10.3 $mM^{-1}cm^{-1}$; for Fe (III) PTP IX Abs_{535nm} ε= 14.5 mM^{-1} cm^{-1}, for Zn(II) PTP IX Abs_{550nm} ε=12.7 $mM^{-1}cm^{-1}$).

<u>*Preparation and characterization of Mn-, Fe- and Zn- SA adducts.*</u>

Mn (III) PTP IX-, Fe (III) PTP IX-, and Zn (II) PTP IX- SA adducts were prepared by slowly addition of metal heme hydroxide (1N KOH solution) to a BSA phosphate-buffered solution (PBS: NaCl 0.138 M, KCl 2.7 mM, K_2HPO_4 8.6 mM and KH_2PO_4 1.5 mM). In all the experiments the concentration of BSA was always greater than the concentration of the metal heme (1.2 mol BSA / 1 mol metal PTP IX) in order to avoid free heme. pH of the solution was checked and adjusted to be 7.2 ± 0.1. Solutions were gentle stirred for 10 min at room temperature. After the preparation, the specimens were filtered by using a 0.2 µm nitrocellulose filter and stored at 4°C.

The assessment of Mn (III) PTP IX-, Fe (III) PTP IX-, and Zn (II) PTP IX binding to BSA was carried by recording UV-visible absorption spectra (for Mn (III) PTP IX-SA $Abs_{466\ nm}$ ε= 28 mM^{-1} cm^{-1}; for Fe (III) PTP IX-SA $Abs_{402\ nm}$ ε= 107 $mM^{-1}cm^{-1}$, for Zn (II) PTP IX-SA $Abs_{415\ nm}$ ε= 298 $mM^{-1}cm^{-1°}$. Absorbance was measured by using a 6715 UV-Vis Spectrophotometer Jenway (Bibby Scientific Limited, Beacon Road, Stone, Staffordshire, ST15 OSA, UK).

Relaxometric quantification of metal concentration

A further check about the exact concentration of the metal in the Mn (III) -SA and Fe (III) -SA specimens were determined by using a ^{1}H relaxometric approach. Briefly, 70 µL of specimens were added to an equal volume of 65% nitric acid and placed into sealed glass vials O.N. 120°C.

Then, water proton $1/T_1$ ($=R_1$) longitudinal relaxation rate of acidic solutions was measured at 25°C by using a Stelar SpinMaster relaxometer and concentration of metal was calculated on the basis of previously obtained calibration curves of iron or manganese acqua-ion solutions.

Cells

Murine macrophages J774A.1 (TIB-67™) cells were purchased from ATCC®. TS/A murine breast cancer cells were derived at the University of Torino from a spontaneous mammary adenocarcinoma which arose in a retired breeder BALB/c female.[1]

J774A.1 cells and TS/A cells were cultured in Dulbecco Minimum Essential Medium (DMEM) supplemented with 10% fetal bovine serum, 2 mM glutamine, 100 U/mL penicillin, and 100 µg/mL streptomycin.

TS/A cells were cultured in RPMI (Roswell Park Memorial Institute)[1064] medium supplemented with 10% heat-inactivated fetal bovine serum (FBS), 2 mM glutamine, 100 U/ml penicillin and 100 µg/ml streptomycin.

Cells were seeded in 75-cm^2 flasks at a density of *ca*. 2 x 10^4 cells/cm^2 in a humidified 5% CO_2 incubator at 37°C. At confluence, they were detached by adding 1 mL of a trypsin-EDTA solution (0.25% *wt/vol* trypsin, 0.53 mM EDTA).

All cell lines were negative for mycoplasma, as tested using the MycoAlert Mycoplasma Detection Kit (Lonza Sales AG-EuroClone S.p.A., Milan, Italy).

All cell media and supplements were purchased from Lonza Sales AG-EuroClone S.p.A. (Milan, Italy). Tests to assess the toxicity of Mn-, Fe- or Zn-SA preparations were carried out by using MTT assay on the two cell lines. [2] Cells were seeded into 96-well tissue culture plates (1 x 10^4 TS/A cells *per* plate and 1.5x 10^4 J774A.1 cells *per* plate) 24 hours before the experiment in presence of the proper cell media, in humidified 5% CO_2 incubator at 37°C.

Then, they were incubated for 4 h, with fresh complete medium in the presence of Mn-, Fe- or Zn-SA adducts (concentration is 0 ÷ 0.6 mM). After incubation, the medium was removed, and the cells were washed and reincubated in the presence of fresh medium supplemented with 0.5 mg/mL MTT (thiazolyl blue tetrazolium bromide, Sigma Aldrich) for 4 hours in a humidified 5% CO_2 incubator at 37°C. Then, the MTT solution was removed, and the plates were filled with DMSO (0.1 mL for plate) for 1/2 hour at room temperature, under gentle agitation, to allow the formazan crystals to solubilize. The absorbance of the resulting-coloured solutions was quantified using a 96-multiwell Glomax Promega Microplate Reader (λ=560 nm). The percentage of viable cells was calculated based on the control blank cells using the following formula:

$$\text{Viable cells } \% = \frac{\text{Abs - T}}{\text{Abs - cnt}} \times 100$$

where Abs-T is the mean absorbance of the treated cells, and Abs-cnt is the mean absorbance of the control untreated cells (after subtraction of the absorption of empty plates as background). Cell experiments were repeated in quadruplicate, and the data are reported as mean ± standard deviation. The blank was repeated 10 times.

Animal models

The *in vivo* experiments were performed on male 10 weeks Balb/c mice (Charles Rivers Laboratories, Inc.), weight of 24 ± 2 g. The mice were kept in standard housing with standard rodent chow, water available *ad libitum*, and a 12-hour light/dark cycle. Experiments were performed according to the Amsterdam Protocol on Animal Protection, in conformity with institutional guidelines that are in compliance with national laws (D.L.vo 116/92, D.L.vo 26/2014, and following additions) and international laws and policies (2010/63/EU, EEC Council Directive 86/609, OJL 358, Dec 1987; NIH Guide for the Care and Use of Laboratory Animals, US National Research Council, 1996).

For the tumour model preparation and PAI experiments, mice were anesthetized via the intramuscular injection of tiletamine/zolazepam (Zoletil 100; Virbac, Milan, Italy) 20 mg/kg plus xylazine (Rompun; Bayer, Milan, Italy) 5 mg/kg using a 27-gauge syringe. For the tumour model preparation, *ca*. 3x10^5 TS/A cells were suspended in 0.1mL of PBS and subcutaneously injected in the flank of mice.

PAI measurements

All US and PA images were acquired on a VisualSonics Vevo 2100 LAZR Imaging Station (VisualSonics, Inc., Toronto, Canada) equipped with a LZ250 transducer operating at 21 MHz. The in vitro PAI characterization of MSNs was carried out by preparing a phantom constituted by thin-layer plastic tubes surrounded by 1% agarose gel. Specimens to be analysed were loaded inside capillaries by using a syringe.

An US gel was applied over the region of interest before image acquisition. As reference image, grey scale B-mode US images at high resolution were acquired. This was carried out by using a high-frequency ultrasound probe (MS550D, VisualSonics, Canada, broadband frequency: 22 MHz - 55MHz, image axial resolution: 40 um) operating at 40 MHz. For PA imaging a 21 MHz frequency probe was used, provided with a laser tuneable in the 680-970 nm range. The laser energy was continuously monitored and eventually

adjusted through laser recalibration and optimization, in order to acquire PA images with the same energy. PA spectra were acquired investigating the region 680-970 nm, with 2 nm steps (persistence=4).PA images of Mn-, Fe- or Zn-SA adducts were acquired by setting the proper wavelength (persistence=4).

For *in vivo* experiments, mice were anesthetized as above reported and hairs were removed over the tumour area using a depilatory cream. Tail vein was catheterized for the following *i.v.* injection of Mn-SA. An US gel was applied on the region of interest before image acquisition. US and PA images were acquired as above reported. PAI spectra and PA images of tumour region were acquired before and 5 min after the slow *i.v.* injection of the Mn-SA (0.02 mmol/kg *b.w.*, corresponding to *ca*. 0.2 mM of Mn-SA in the blood, volume of bolus 200 mL). All PA images were co-registered with grey scale B-mode imaging. ROIs were manually drawn, and PA signal intensity measured.

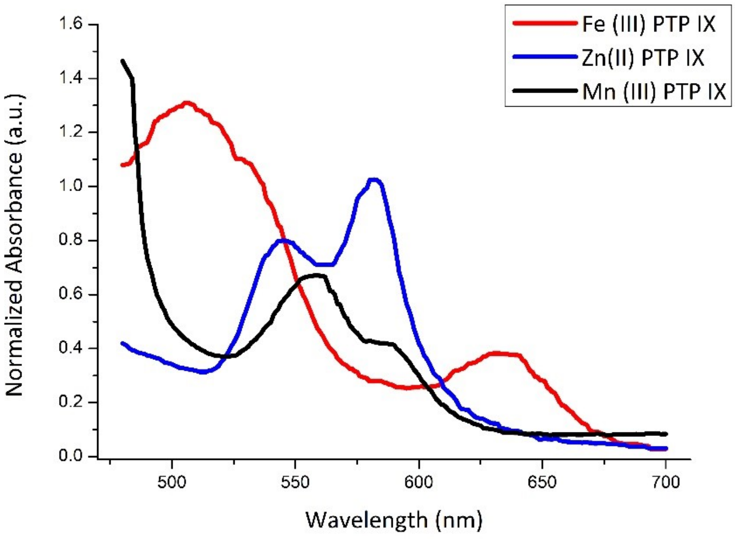

Figure S1 Absorption spectra for the Mn (III), Fe (III) and Zn (II) – protoporphyrin IX -dissolved in KOH 1N and added to a 10% (w/v) SDS solution in 1M Imidazole.

Supporting results

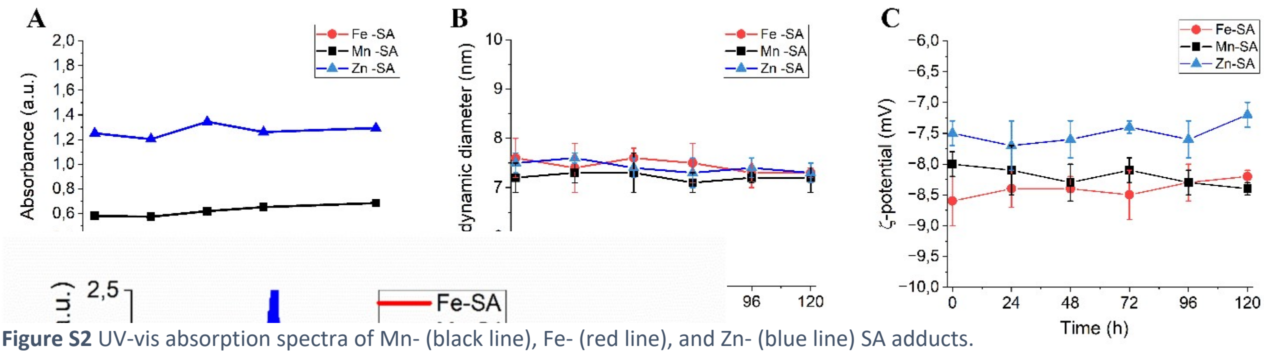

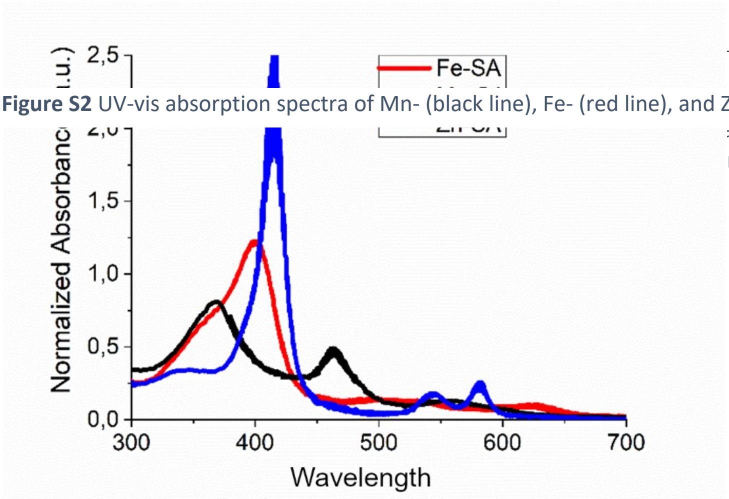

Figure S2 UV-vis absorption spectra of Mn- (black line), Fe- (red line), and Zn- (blue line) SA adducts.

[illegible] absorbance at 37 °C, under agitation (λ =. 402 nm for [illegible] ment of size and (C) DLS measurement of ζ-potential.

Supporting references

1. Nanni P, de Giovanni C, Lollini PL, Nicoletti G, Prodi G. TS/A: a new metastasizing cell line from a BALB/c spontaneous mammary adenocarcinoma. *Clin Exp Metastasis*. **1983**, 1: 373-80. doi: 10.1007/BF00121199.
2. Liu X, Rodeheaver DP, White JC, Wright AM, Walker LM, Zhang F, Shannon S. A comparison of in vitro cytotoxicity assays in medical device regulatory studies. *Regul Toxicol Pharmacol*. **2018**, 97: 24-32. doi: 10.1016/j.yrtph.2018.06.003.
